# Synaptic Plasticity at Inhibitory Synapses in the Ventral Tegmental Area Depends upon Stimulation Site

**DOI:** 10.1523/ENEURO.0137-19.2019

**Published:** 2019-11-13

**Authors:** Robyn St. Laurent, Julie Kauer

**Affiliations:** Department of Psychiatry and Behavioral Sciences, Stanford University School of Medicine, Stanford, CA 94305

**Keywords:** dopamine, electrophysiology, GABA, midbrain, potentiation, synaptic plasticity

## Abstract

Drug exposure induces cell and synaptic plasticity within the brain reward pathway that could be a catalyst for progression to addiction. Several cellular adaptations have been described in the ventral tegmental area (VTA), a central component of the reward pathway that is the major source of dopamine release. For example, administration of morphine induces long-term potentiation (LTP) of excitatory synapses on VTA dopamine cells and blocks LTP at inhibitory synapses. Drug-induced synaptic changes have a common endpoint of increasing dopamine cell firing and dopamine release. However, gaining a complete picture of synaptic plasticity in the VTA is hindered by its complex circuitry of efferents and afferents. Most studies of synaptic plasticity in the VTA activated a mixed population of afferents, potentially yielding an incomplete and perhaps misleading view of how drugs of abuse modify VTA synapses. Here, we use midbrain slices from mice and find that electrical stimulation in two different regions induces different forms of plasticity, including two new forms of LTP at inhibitory synapses. High-frequency stimulation (HFS) induces LTP independently of NMDA receptor (NMDAR) activation, and surprisingly, some inhibitory inputs to the VTA also undergo NMDAR-independent LTP after a low-frequency stimulation (LFS) pairing protocol.

## Significance Statement

Synaptic plasticity of inhibitory inputs onto dopamine cells in the ventral tegmental area (VTA) has a major influence on the circuits implicated in addictive behaviors. The location of electrical stimulation in an acute midbrain slice dictated the response of inhibitory inputs to plasticity induction protocols. We describe a new form of synaptic strengthening that occurs at an opioid-sensitive input to the VTA.

## Introduction

The ventral tegmental area (VTA) contains dopaminergic cells that receive inhibitory innervation from GABAergic cell bodies originating within the VTA and from many other brain regions (Watabe-Uchida et al., 2012; Beier et al., 2015). Despite a wealth of anatomical and behavioral studies investigating the diversity of VTA afferents, plasticity at inhibitory synapses was historically described without identification of the presynaptic partner (Melis et al., 2002; Liu et al., 2005; Nugent et al., 2007, 2009; Niehaus et al., 2010; Dacher and Nugent, 2011; Padgett et al., 2012; Graziane et al., 2013; Kodangattil et al., 2013; Polter et al., 2014). For example, nitric-oxide-dependent long-term potentiation (LTP_GABA_) can be triggered using electrical stimulation within the VTA (Nugent et al., 2007); however, when specific afferents were isolated using optogenetics, induction of LTP_GABA_ was found to depend on the presynaptic partner (Simmons et al., 2017; Polter et al., 2018). Specifically, LTP_GABA_ is expressed at nucleus accumbens and VTA GABA_A_ synapses, but not rostromedial tegmental nucleus (RMTg)-originating GABA_A_ synapses. These observations demonstrate that all GABAergic synapses cannot be assumed to share a common plasticity mechanism. The idea that plasticity is segregated to specific populations is not a new one, and in fact many reports segregate experiments by postsynaptic cell identity. For example, long-term depression (LTD) induced by low-frequency afferent stimulation is only expressed in putative dopamine cells in the VTA that express large H currents (I_h_; Dacher and Nugent, 2011). With local electrical stimulation in acute slices, it is possible to isolate synapses of one neurotransmitter type pharmacologically, but the identity of the presynaptic source is not always as easy to manipulate or determine. Although the location of the postsynaptic VTA cell (e.g., medial vs lateral VTA) can sometimes predict output site, inputs from over 20 brain regions contact dopamine cells in all VTA subregions (Beier et al., 2015). It is possible that other plasticity mechanisms have yet to be uncovered because their expression is limited to a subset of inputs, and therefore not apparent with global activation of all inputs. Here, we use different electrical stimulation sites, and report two ways of inducing LTP at inhibitory synapses in the VTA with a mechanism(s) that is non-overlapping with that of LTP_GABA_ or other known forms of LTP at inhibitory synapses in the VTA.

## Materials and Methods

### Animals

All procedures were conducted in accordance with the guidelines of the National Institutes of Health for animal care and use and were approved by the Brown University and Stanford University Institutional Animal Care and Use Committees. This study used VGAT::IRES-Cre (RRID:IMSR_JAX:028862, strain code: B6J.129S6(FVB)-Slc32a1^tm2(cre)Lowl^), DAT::IRES-Cre (RRID:IMSR_JAX:006660, strain code: B6.SJL-Slc6a3^tm1.1(cre)Bkmn/J^; Zhuang et al., 2005), Ai14 Cre-reporter mice (RRID:IMSR_JAX:007908, strain code: B6;129S6-Gt(ROSA)26Sor^tm14(CAG-tdTomato)Hze/J^), VGAT-ChR2(H134R)-EYFP (RRID:IMSR_JAX:014548, strain code: B6.Cg-Tg(Slc32a1-COP4*H134R/EYFP)8Gfng/J; (Zhao et al., 2011), and C57BL/6 (RRID:IMSR_JAX:000664) male and female mice bred in-house. Mice were maintained on a 12/12 h light/dark cycle and provided food and water *ad libitum*.

### Preparation of brain slices

Horizontal brain slices (220 µm) were prepared from deeply anesthetized mice. Briefly, anesthetized mice were perfused with ice-cold oxygenated artificial CSF (ACSF): 126 mM NaCl, 21.4 mM NaHCO_3_, 2.5 mM KCl, 1.2 mM NaH_2_PO_4_, 2.4 mM CaCl_2_, 1.0 mM MgSO_4_, 11.1 mM glucose, and 5 mM sodium ascorbate. Following perfusion, the brain was rapidly dissected and horizontal slices (220 μm) were prepared using a vibratome. Slices recovered for 1 h at 34°C in oxygenated HEPES holding solution: 86 mM NaCl, 2.5 mM KCl, 1.2 mM NaH_2_PO_4_, 35 mM NaHCO_3_, 20 mM HEPES, 25 mM glucose, 5 mM sodium ascorbate, 2 mM thiourea, 3 mM sodium pyruvate, 1 mM MgSO_4_, and 2 mM CaCl_2_ (Ting et al., 2014), and then were held in the same HEPES solution at room temperature until use. Slices were then transferred to a recording chamber where they were submerged in ACSF without sodium ascorbate.

### Electrophysiology

Electrophysiological experiments were performed in horizontal midbrain slices containing the VTA that were continuously perfused with ACSF containing 10 µM 6,7-dinitroquinoxaline-2,3-dione (DNQX) and 1 μM strychnine, AMPA and glycine receptor antagonists respectively. Except where noted, recordings also included the NMDA receptor (NMDAR) antagonist D-APV (50 or 100 µM). Whole-cell recordings were performed from neurons in the lateral VTA with KCl pipette solution and voltage-clamped at –70 mV. Patch pipettes were filled with the following: 125 mM KCl, 2.8 mM NaCl, 2 mM MgCl_2_, 2 mM ATP-Na^+^, 0.3 mM GTP-Na^+^, 0.6 mM EGTA, and 10 mM HEPES. In some experiments, EGTA was increased to 15 mM, or GDP-β-S (1 mM) was included in the pipette solution as noted. The presence of a large hyperpolarization-activated inward current (I_h_) was used to select postsynaptic cells for recording, although we are aware that this metric can allow inclusion of a subset of non-dopamine neurons. If the steady-state I_h_ was >25 pA during a step from –50 to –100 mV, the cell was included in analyses. In a subset of recordings, dopamine cells were also identified via fluorescence imaging using a DAT::IRES-cre x TdTomato reporter line. All experiments were performed at 30°C, maintained by an automatic temperature controller. The series resistance was monitored continuously during the experiment and cells were discarded for deviations >15%.

### Stimulation protocols

**Figure 1.**
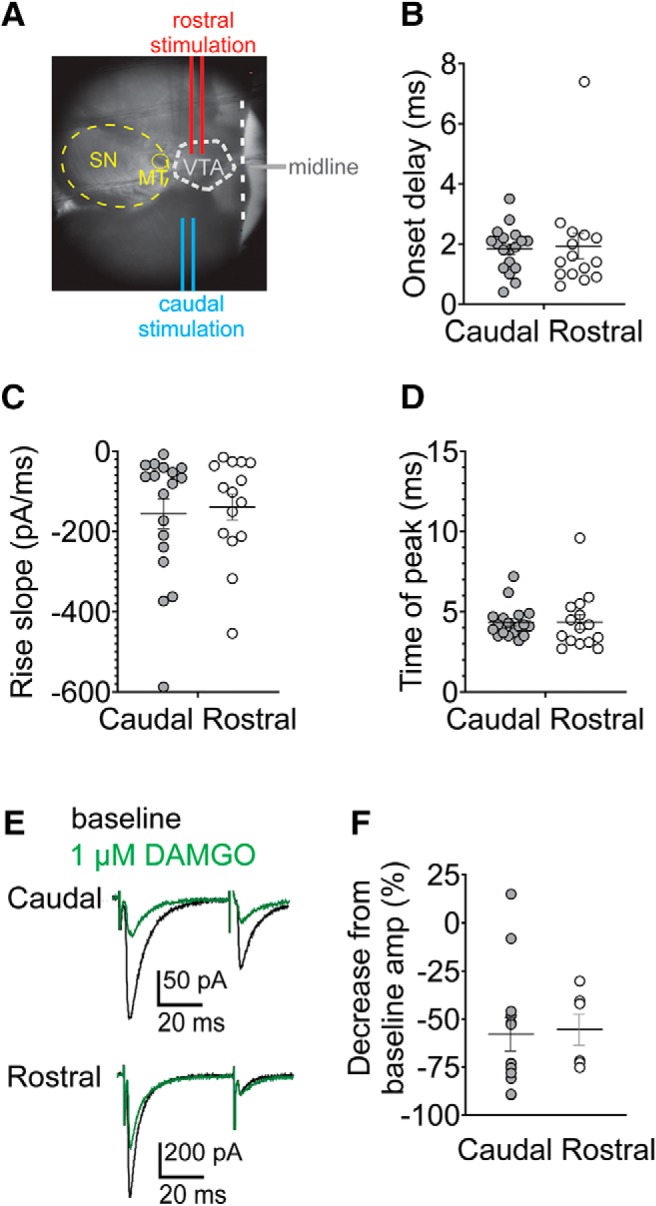
Electrical stimulation in horizontal midbrain slices. ***A***, Recording setup illustrating caudal or rostral placements of the bipolar stimulating electrodes. Analysis of caudal versus rostral IPSC: onset delay (***B***), rise slope (***C***), and time to peak amplitude (***D***). ***E***, Example IPSCs illustrating control IPSCs (black) and in the μ-opioid receptor agonist, DAMGO (1 μM; green), for caudal or rostral inputs. ***F***, Mean IPSC amplitude depression after DAMGO (1 µM), for each input. Error bars represent SEM.

For electrical stimulation, a bipolar stainless-steel stimulating electrode was placed caudal to the VTA ∼500 μm from the recorded cell; for rostral placement the stimulating electrode was placed within the VTA at 200–500 μm from the recorded cell (Fig. 1*A*). IPSCs were evoked at 0.1 Hz using 100-μs current pulses. We used input-output curves to identify the stimulation intensity used for plasticity experiments for both rostral and caudal afferents, and the baseline amplitude was at 50% of this generated curve. No correlation was observed between stimulation intensity and LTP magnitude. This stimulation protocol did not produce action potentials escaping voltage-clamp, but in the rare cases that cells began spiking later in the recording, they were excluded from analysis. Channelrhodopsin-induced synaptic currents were evoked at 0.033 Hz using 0.1- to 5-ms light pulses from a white LED (Mightex) controlled by driver (ThorLabs) and reflected through a 40× water immersion lens. When feasible, IPSCs were shown to be GABA_A_ receptor mediated by bath application of 10 µM bicuculline at the end of recordings. For all stimulus frequencies, intensity remained constant throughout the experiment.

### Statistical analysis

Results are expressed as mean ± SEM. Significance was determined using a two-tailed paired Student’s *t* test or one-way ANOVA with significance level of *p* < 0.05 (Table 1). LTP values are reported as averaged IPSC amplitudes for 10 min just before LTP induction compared with averaged IPSC amplitudes during the 10-min period from 10–20 min after manipulation. Paired-pulse ratios (50-ms interstimulus interval) and coefficient of variation were measured over 10-min epochs of 10–30 IPSCs each. The paired pulse ratio was calculated using the average value for all IPSC2 amplitudes divided by the average value for the corresponding IPSC1 amplitudes and reported as the mean paired pulse ratio for that epoch. 1/CV^2^ values were determined by dividing the mean amplitude of IPSCs squared recorded over 10-min epochs by the mean variance of these IPSCs.

### Materials

DNQX was obtained from Sigma-Aldrich. D-2-amino-5-phosphonopentanoic acid (APV), bicuculline, [D-Ala^2^, *N*-Me-Phe^4^, Gly-ol^5^]-enkephalin (DAMGO), forskolin, and naloxone were obtained from Tocris. Strychnine was obtained from Tocris or Abcam.

## Results

### Location of electrical stimulation determines expression of synaptic plasticity

Most reports examining synaptic plasticity in the VTA have used a stimulating electrode placed within the VTA 200–500 µM rostral to the recorded cell in a horizontal slice (Fig. 1*A*). This approach has been assumed to randomly sample the synaptic inputs onto cells within the VTA. We hypothesized that stimulating caudal to and outside of the VTA, ∼500 µM from the recorded cell, might bias the inputs differently than with a rostral placement (Fig. 1*A*). We refer to this as “caudal” stimulation. We recorded IPSCs in putative dopamine cells identified by a large I_h_ and compared synaptic properties using either caudal or rostral electrical stimulation. We did not detect any differences between IPSCs evoked by rostral or caudal stimulation in the onset delay, rise slope, or time of peak amplitude of IPSCs (Fig. 1*B–D*; paired *t* test of rostral versus caudal onset delay: *p* = 0.82 ^a^; rise slope: *p* = 0.74^b^; time of peak: *p* = 0.98^c^; rostral: *n* = 15 cells, caudal: *n* = 18 cells). Opioids depress GABAergic inhibition in the VTA; therefore, we compared the opioid-sensitivity of caudal and rostral-stimulated IPSCs. IPSCs from both stimulating locations were depressed by 1 µM DAMGO to the same degree (Fig. 1*E*,*F*; rostral = –55 ± 8%, *n* = 6 cells; caudal = –58 ± 9%, *n* = 13 cells; rostral versus caudal: *p* = 0.86^d^). Thus, synaptic properties were similar when stimulating either the rostral or caudal site.

**Figure 2.**
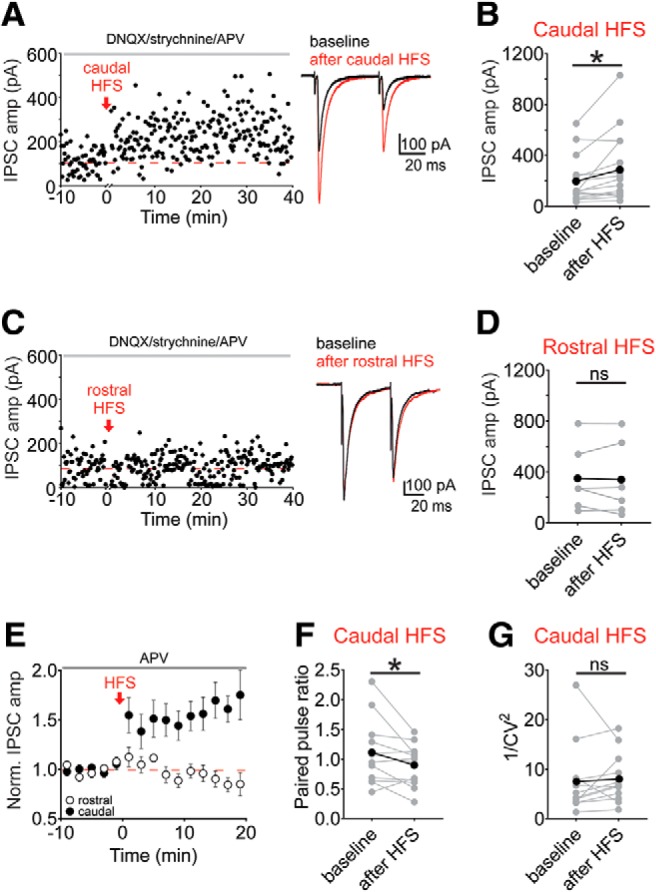
Location of electrical stimulation determines expression of synaptic plasticity. ***A***, Representative experiment showing LTP induction by HFS with a caudal electrode placement. Inset, Baseline (black traces) and 10–20 min after HFS (red traces). ***B***, Mean IPSC amplitudes from a 10-min baseline and 10–20 min after caudal HFS (*n* = 16 cells). In this and subsequent figures, thicker black symbols/lines represent the mean response across all cells. ***C***, Representative experiment with HFS of a rostral electrode. Inset, Baseline (black traces) and 10–20 min after HFS (red traces). ***D***, Mean IPSC amplitudes from a 10-min baseline to 10–20 min after rostral HFS (*n* = 6 cells). ***E***, Time course of averaged IPSC amplitudes before and after HFS (closed symbols = caudal, *n* = 16; open symbols = rostral, *n* = 6). ***F***, Paired pulse ratios before and after caudal HFS from each cell that potentiated >10% of basal values (*n* = 12 cells). ***G***, 1/CV^2^ values before and after caudal HFS from each cell that potentiated >10% of basal values (*n* = 12 cells); *p < 0.05, paired *t* test of amplitude of 10-min baseline versus 10–20 min after HFS, ns, not significant. Error bars represent SEM.

We next used a stimulation protocol known to induce the nitric oxide-dependent LTP_GABA_: high-frequency stimulation (HFS) consisting of two 100-Hz tetani separated by 10 s (Nugent et al., 2007). LTP_GABA_ is dependent on NMDAR activation that leads to the release of nitric oxide and activation of a signaling cascade that increases presynaptic GABA release (Nugent et al., 2007, 2009). Instead, HFS of the caudally stimulated site resulted in LTP, even with the NMDAR antagonist, APV (100 µM), in the bath solution (Fig. 2*A*,*B*,*E*; 157 ± 23% of baseline value; paired *t* test: *p* = 0.018^e^, *n* = 16 cells). Conversely and consistent with prior results, the same tetanus of a rostrally-placed electrode did not potentiate IPSCs in APV (Nugent et al., 2007; Fig. 2*C–E*; 90 ± 11% of baseline value; paired *t* test: *p* = 0.73^f^, *n* = 6 cells). Potentiation after HFS of the caudally-stimulated electrode was correlated with a decrease in paired pulse ratio for cells that potentiated at least 10% (Fig. 2*F*; baseline: 1.1 ± 0.2, after HFS: 0.9 ± 0.1), without a change in 1/CV^2^ values (Fig. 2*G*; baseline: 7.5 ± 2.1, 10–20 min after HFS: 8.1 ± 1.4; paired *t* test: *p* = 0.048^g^ and *p* = 0.70^h^, respectively, *n* = 12 cells). These findings supported our hypothesis that electrode placement may activate different subsets of afferents and lead to a different outcome when performing protocols to induce synaptic plasticity.

### Low-frequency stimulation (LFS) potentiates caudal-evoked inhibitory inputs

**Figure 3.**
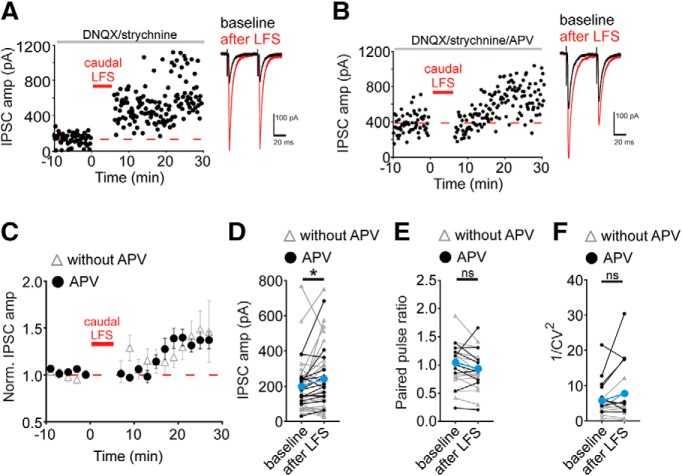
LFS of caudal electrode induces LTP. Representative experiment with LFS with a caudal electrode placement without APV (***A***) or with APV (***B***). Insets, Baseline (black traces) and 10–20 min after LFS (red traces). ***C***, Time course of averaged IPSC amplitudes before and after LFS. ***D***, Mean IPSC amplitudes from a 10-min baseline to 10–20 min after caudal LFS (*n* = 38 cells; without APV, *n* = 25, with APV, *n* = 13). ***E***, Paired pulse ratios before and after caudal LFS from each cell that potentiated >10% of basal values (*n* = 22 cells). ***F***, 1/CV^2^ values before and after caudal LFS from each cell that potentiated >10% of basal values (*n* = 19 cells). ***D–F***, Gray symbols/lines, no APV, black symbols/lines, with APV present; *p < 0.05, paired *t* test of amplitude of 10-min baseline versus 10–20 min after LFS, ns, non significant. Error bars represent SEM.

Inhibitory synapses can be regulated bidirectionally by different afferent stimulation patterns. In an earlier study, LFS of afferents by a stimulating electrode placed rostral to the VTA cell being recorded was used to elicit LTD. LTD is induced by LFS, 6 min of 1-Hz stimulation while voltage clamping the postsynaptic cell at –40 mV (LFS-LTD; Dacher and Nugent, 2011). LFS-LTD occurs independently of NMDAR activation and is partially blocked by a dopamine D2 receptor antagonist (Dacher and Nugent, 2011). Given the surprising result with HFS of a caudally-placed electrode, we asked whether LFS of a caudally-placed stimulating electrode would also induce LTD. Instead, LFS of caudally-evoked IPSCs triggered LTP both in the absence or presence of APV (LFS-LTP_GABA_; Fig. 3*A–D*; 131 ± 10% of baseline value; *p* = 0.026^i^, *n* = 38 cells). PPR was not significantly altered in cells potentiating by at least 10% after LFS (Fig. 3*E*; baseline: 1.0 ± 0.1, 10–20 min after LFS: 0.9 ± 0.1; *p* = 0.058^j^, *n* = 22 cells) and neither were the normalized 1/CV^2^ values (Fig. 3*F*; baseline: 5.7 ± 1.1, 10–20 min after LFS: 7.8 ± 1.8; *p* = 0.09^k^, *n* = 19 cells). This surprising finding led us to conclude that as with HFS-induced LTP, previous observations of LTD following LFS were likely dependent on activation of a subset of VTA afferents.

### LFS of optically-evoked inhibitory inputs in the VTA does not induce plasticity

**Figure 4.**
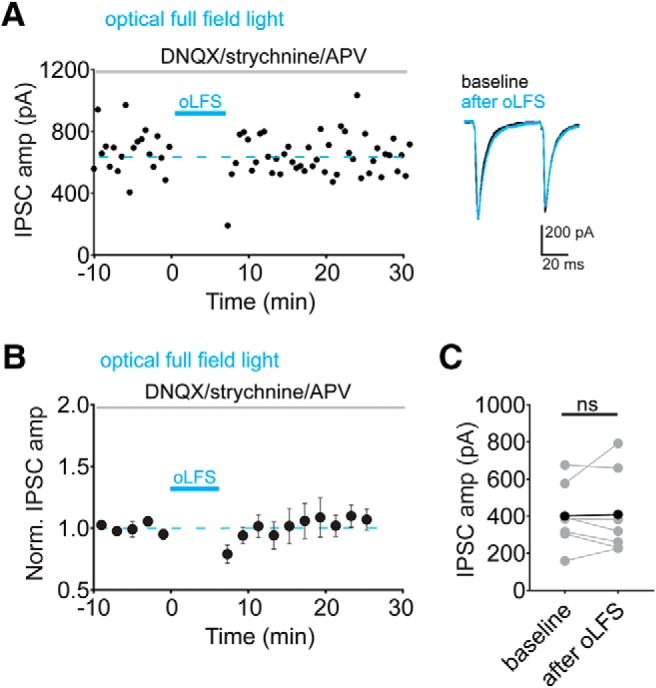
No effect with low-frequency optical stimulation of VGAT^+^ synapses. ***A***, Representative experiment with optical LFS. Inset, Baseline (black traces) and 10–20 min after LFS (red traces). ***B***, Time course of averaged IPSC amplitudes before and after LFS (*n* = 7). ***C***, Mean IPSC amplitudes from a 10-min baseline to 10–20 min after optical LFS (*n* = 7 cells). Error bars represent SEM, ns, not significant.

There are many sources of GABAergic inhibition in the VTA and given that LFS can induce either LTD or LTP, depending on stimulation site, we wondered which form of plasticity was predominant when activating GABAergic synapses more globally. We hypothesized that just a subset of VTA synapses express LFS-LTP_GABA_, so that when using optical stimulation of VGAT^+^ inputs in a VGAT-ChR2 transgenic mouse line, both forms of plasticity might occur at different synapses on the same dopamine cell. We used a BAC transgenic mouse line, VGAT-ChR2(H134R)-EYFP, to activate multiple GABAergic inputs in the VTA (Zhao et al., 2011), and used whole-field LED illumination of the slice to activate inhibitory inputs. After generating a stable 10-min baseline of light-evoked IPSCs, we delivered optical LFS while depolarizing the postsynaptic cell to –40 mV. In contrast to what we observed with caudal electrical stimulation, the mean light-evoked IPSC amplitude was unchanged after optical LFS (Fig. 4*A–C*; 102 ± 10% of baseline value; *p* = 0.83^l^, *n* = 7 cells). PPR was not significantly altered after LFS (data not shown; baseline: 0.72 ± 0.07, 10–20 min after LFS: 0.72 ± 0.05, *p* = 0.97^m^, *n* = 7 cells) and neither were 1/CV^2^ values (data not shown; baseline: 20.0 ± 2.4, 10–20 min after LFS: 20.2 ± 3.6, *p* = 0.95^n^, *n* = 7 cells).

### Forskolin potentiation does not occlude LFS-induced LTP

**Figure 5.**
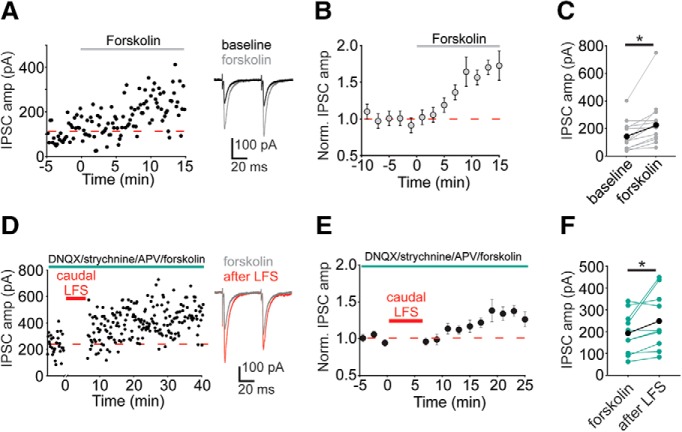
Forskolin potentiates GABAergic synapses evoked with caudal stimulation but does not prevent subsequent potentiation by caudal LFS. ***A***, Representative experiment with 10 µM forskolin. Inset, Baseline (black traces) and in forskolin (gray traces). ***B***, Time course of averaged IPSC amplitudes before and during forskolin. ***C***, Mean IPSC amplitudes from a 10-min baseline to 10–20 min after forskolin addition (*n* = 14 cells). ***D***, Representative experiment with caudal LFS after potentiation by 10 µM forskolin. Inset: baseline in forskolin (gray traces) and 10–20 min after LFS (red traces). ***E***, Time course of averaged IPSC amplitudes before and after caudal LFS after forskolin-induced potentiation was established. ***F***, Mean IPSC amplitudes from a 10-min baseline to 10–20 min after forskolin (*n* = 10 cells); *p < 0.05, paired *t* test of amplitude of 10-min baseline versus 10–20 min after forskolin or LFS. Error bars represent SEM.

Forskolin is known to potentiate many synapses. Forskolin activates adenylyl cyclase which potentiates GABAergic synapses in the VTA (Melis et al., 2002; Nugent et al., 2009) as well as at many excitatory and inhibitory synapses throughout the CNS (Briggs et al., 1988; Greengard et al., 1991; Cameron and Williams, 1993; Chavez-Noriega and Stevens, 1994; Huang and Kandel, 1994, 1998; Weisskopf et al., 1994; Bonci and Williams, 1996; Salin et al., 1996; Bonci and Williams, 1997; Castro-Alamancos and Calcagnotto, 1999; Linden and Ahn, 1999; Mellor et al., 2002). Both prior VTA studies using forskolin and stimulating GABAergic VTA afferents used rostral electrical stimulation. We wondered whether forskolin would also potentiate inputs evoked with caudal afferent stimulation or whether these synapses would prove distinct again. We found that 10 μM forskolin potentiated IPSCs stimulated with a caudally-placed electrode (Fig. 5*A–C*; 183 ± 19% of baseline value; *p* = 0.003^o^, *n* = 14 cells), although PPR was not significantly altered after forskolin (data not shown; baseline: 0.9 ± 0.1, 10–20 min after LFS: 0.9 ± 0.1; *p* = 0.65^p^, *n* = 13 cells). Forskolin potentiation occludes NMDAR-dependent LTP_GABA_ (Nugent et al., 2009). Therefore, we performed occlusion experiments to ask if forskolin potentiation occludes caudal LFS-induced LTP. However, forskolin-induced potentiation did not occlude further potentiation by LFS (Fig. 5*D–F*; 131 ± 10% of baseline value; *p* = 0.046^q^, *n* = 10 cells). These data suggest that the mechanism underlying LFS-induced LTP is distinct from that of forskolin potentiation.

### LFS-induced LTP does not require postsynaptic G-protein-coupled receptor (GPCR) signaling and is not prevented by postsynaptic EGTA

**Figure 6.**
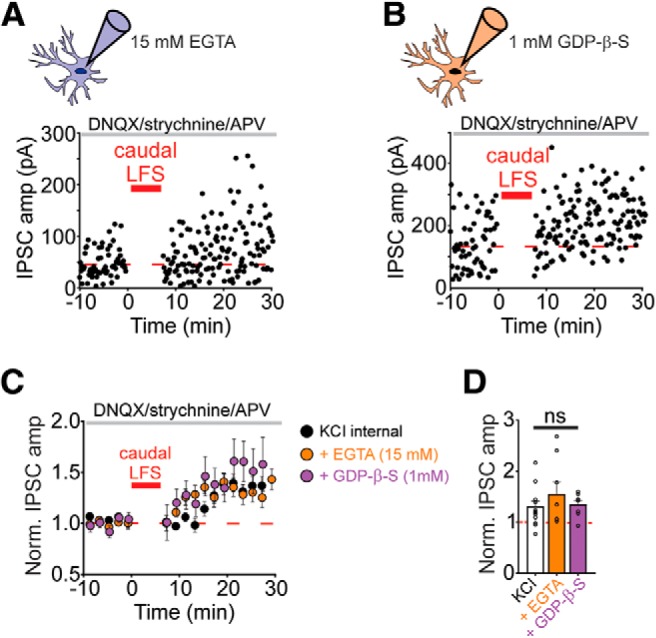
LFS-induced LTP does not require postsynaptic calcium elevation or GPCR activation. ***A***, Representative experiment with caudal LFS when 15 mM EGTA was included in the patch pipette. Inset, Baseline (black traces) and 10–20 min after LFS (red traces). ***B***, Representative experiment with caudal LFS when 1 mM GDP-β-S was included in the patch pipette intracellular solution. Inset, Baseline (black traces) and 10–20 min after LFS (red traces). ***C***, Time course of averaged IPSC amplitudes before and after caudal LFS with: normal KCl internal solution (black symbols, *n* = 14), or with 15 mM EGTA (orange symbols, *n* = 8) or 1 mM GDP-b-S (purple symbols, *n* = 8) in the pipette solution. ***D***, Mean IPSC amplitudes normalized to a 10 minute baseline period at 10–20 min after LFS with the different internal solutions listed in ***C***. Error bars represent SEM, ns, not significant.

Potentiation after LFS was not associated with a significant change in the paired pulse ratio or coefficient of variation (Fig. 3*E*,*F*), suggesting that the mechanism may reflect an increased GABAergic sensitivity of the postsynaptic cells. Most forms of LTP are triggered by increases in calcium concentration in the postsynaptic cell, and so we tested whether LFS-LTP requires a rise in postsynaptic calcium. When the concentration of the calcium chelator, EGTA, was raised to 15 mM in the patch pipette, LFS still resulted in potentiation of caudally-evoked IPSCs (Fig. 6*A,C,D*; 155 ± 24% of baseline value; *n* = 8 cells). An alternative postsynaptic mechanism might require activation of receptors on the postsynaptic cell other than GABA_A_ receptors. For example, the report of LFS-LTD found that depression was partially dependent on dopamine D2 receptors (Dacher and Nugent, 2011), which are coupled to the G_i_ subtype of GPCRs. When we included an inhibitor of GPCR activity (1 mM GDP-βS) in the patch pipette to block all postsynaptic GPCR signaling, LFS still potentiated caudally-evoked IPSCs (Fig. 6*B,C,D*; 135 ± 8% of baseline value; *n* = 8 cells). The magnitude of LTP after LFS was not significantly different for high EGTA or GDP-β-S conditions than experiments with normal KCl internal solution (*F*_(2,27)_ = 0.77, *p* = 0.48^r^, *n* = 8 cells high EGTA, *n* = 8 cells GDP-β-S, *n* = 14 cells normal KCl). Together our results suggest a mechanism that does not require postsynaptic GPCRs or Ca^2+^ influx. Future experiments will be needed to understand this novel form of LTP.

**Table 1.** Statistical table

Data structure	Type of test	95% confidence interval
^a^Normal distribution	Two-tailed unpaired *t* test	–0.79 to 0.98
^b^Normal distribution	Two-tailed unpaired *t* test	–85.83 to 119.6
^c^Normal distribution	Two-tailed unpaired *t* test	–0.99 to 1.01
^d^Normal distribution	Two-tailed unpaired *t* test	–27.21 to 32.06
^e^Normal distribution	Two-tailed paired *t* test	18.20 to 162.8
^f^Normal distribution	Two-tailed paired *t* test	–78.82 to 59.01
^g^Normal distribution	Two-tailed paired *t* test	–0.43 to –0.002
^h^Normal distribution	Two-tailed paired *t* test	–2.47 to 3.56
^i^Normal distribution	Two-tailed paired *t* test	5.89 to 85.91
^j^Normal distribution	Two-tailed paired *t* test	–0.17 to 0.0029
^k^Normal distribution	Two-tailed paired *t* test	–0.38 to 4.43
^l^Normal distribution	Two-tailed paired *t* test	–87.36 to 104.7
^m^Normal distribution	Two-tailed paired *t* test	–0.15 to 0.15
^n^Normal distribution	Two-tailed paired *t* test	–9.54 to 10.06
^o^Normal distribution	Two-tailed paired *t* test	37.01 to 147.8
^p^Normal distribution	Two-tailed paired *t* test	–0.21 to 0.13
^q^Normal distribution	Two-tailed paired *t* test	1.22 to 110.4
^r^Normal distribution	One-way ANOVA	High EGTA 99.09–210.7; GDP-β-S 115.9–153.4

## Discussion

Most reports describing synaptic plasticity in the VTA used electrical stimulation, which can miss the possibility of circuit specificity that can now be probed using optogenetic tools. For example, LTP_GABA_ (Nugent et al., 2007) was found to vary depending on presynaptic source (Simmons et al., 2017; Polter et al., 2018). Here, we report that inhibitory synapses in the VTA have different requirements for inducing LTP depending on the placement of the stimulating electrode.

### Synaptic plasticity induction

Using the same stimulation protocol but with the electrode at a site that deviated from the usual placement, we serendipitously discovered that we could induce NMDAR-independent LTP using either HFS or LFS. Pairing postsynaptic cell depolarization with afferent stimulation, to substitute for a strong tetanus, is a classic approach used to induce LTP in the hippocampus (Nicoll, 2017). However, this method for inducing LTP is generally due to NMDAR activation. Instead, robust caudal LFS-induced LTP in the VTA was elicited in the presence of an NMDAR antagonist. Previously described LFS-LTD in the VTA is also NMDAR independent (Dacher and Nugent, 2011). How might the same pattern of afferent stimulation result in opposite synaptic plasticity outcomes? One likely explanation is that different electrode locations preferentially activate different subsets of afferents in the VTA slice. The VTA dopamine cells are innervated both by local GABA neurons and by GABA projections originating in regions throughout the brain that may differ in protein expression leading to different forms of synaptic plasticity. Another possibility is that the timing of inputs differs when using the two stimulating electrode locations; however, we did not observe a significant difference in onset delay, rise slope, or time of peak amplitude of IPSCs from caudal versus rostral. We speculate that LFS with mild depolarization may lead to release of a signaling molecule from the postsynaptic or presynaptic cell. If different synapses express different receptor subtypes for that signaling molecule, then release via LFS could result in distinct synaptic strength changes. Future experiments will be needed to determine whether HFS-LTP and LFS-LTP result, e.g., from activation by metabotropic glutamate, endocannabinoid, or dopamine receptors.

### LFS and synaptic plasticity

Numerous studies have shown that LFS induces LTD, often when paired with modest postsynaptic depolarization (Bear and Malenka, 1994; Gutlerner et al., 2002). There are fewer instances where LFS induces LTP, and these generally required pairing with strong depolarization to activate NMDARs (Bonci and Malenka, 1999; Lanté et al., 2006; Ikeda et al., 2007; Dringenberg et al., 2014). Here, we found that LFS potentiates VTA GABA synapses with mild depolarization and LFS that did not require NMDAR activation, an apparently rare mechanism at CNS synapses. One other example is at the excitatory synapse from lateral perforant path to dentate gyrus cells, where LFS also potentiates synapses independently of NMDAR activation (Gonzalez et al., 2014). However, to our knowledge, ours is the first report of LTP elicited by LFS at GABAergic synapses.

### GABAergic afferents innervating the VTA

Exposure to drugs of abuse causes LTP at excitatory synapses on VTA DA cells (Ungless et al., 2001; Saal et al., 2003). Many drugs of abuse also block LTP_GABA_ in the VTA (Nugent et al., 2007; Guan and Ye, 2010; Niehaus et al., 2010; Graziane et al., 2013). The net result of these drug-induced changes in synaptic strength is thought to be increased dopamine cell firing via enhanced excitatory drive and disinhibition. However, if more types of synaptic plasticity exist than previously suspected, differential effects of drugs of abuse on VTA afferents may produce a more nuanced effect on dopamine cell firing. LTP_GABA_ is expressed at VTA_GABA_→VTA but not at RMTg_GABA_→VTA synapses; by analogy, it is likely that the HFS-induced and LFS-induced LTP we report here are expressed only at a subset of inputs. It is difficult to be certain precisely which afferents are sufficiently close to the caudal stimulation site to be activated by our stimulus protocol. Regions other than the RMTg that are located caudal to the VTA with reported GABAergic innervation include: the dorsal raphe, periaqueductal gray, pedunculopontine nucleus, and laterodorsal tegmentum (Beier et al., 2015; Omelchenko and Sesack, 2010; Faget et al., 2016; Ntamati et al., 2018). Given the placement of the caudal electrode in our experiments, it is possible that the presynaptic source of those inputs is from one of these caudal brain regions, although it is alternatively possible that regions located elsewhere in the brain send projections that pass through the caudal stimulation location.

In conclusion, depending on stimulation site, HFS and LFS can induce LTP at GABAergic synapses in the VTA via a mechanism that does not require NMDAR activation. These results support the recent findings that some forms of plasticity, like LTP_GABA_ (Simmons et al., 2017; Polter et al., 2018), are selectively expressed at some synapses but not others. Together, this points toward a specificity of synaptic plasticity based on presynaptic partner and postsynaptic cell identity. Furthermore, this study highlights the fact that there may be plasticity mechanisms in the VTA still to be identified.

## Synthesis

Reviewing Editor: Pablo Castillo, Albert Einstein College of Medicine Decisions are customarily a result of the Reviewing Editor and the peer reviewers coming together and discussing their recommendations until a consensus is reached. When revisions are invited, a fact-based synthesis statement explaining their decision and outlining what is needed to prepare a revision will be listed below. The following reviewer(s) agreed to reveal their identity: Arianna Maffei, Qing-Song Liu.

Your manuscript was evaluated by two experts in the field. Both reviewers agree on the relevance of your findings and quality of the data, but they also raised concerns regarding the quantification and relatively low power of your results (e.g. low “n's”). In addition, there are a number of technical issues and clarification points that should be easy to address. Below please find their specific comments

Reviewer #1:

The manuscript reports how different forms of inhibitory plasticity may be induced by different inputs to dopaminergic neurons in the VTA. While the manuscript does not identify specific mechanisms underlying such effects, it excludes a variety of signaling cascades that are often engaged in the induction of plasticity, pointing to a novel, yet to be discovered, set of mechanisms for inhibitory plasticity in this region. Overall, the experiments are well designed and the quality of the data is high. I have a few minor comments for the authors:

1- The effect of DAMGO on both rostral and caudal evoked responses is important to show that both inputs are equally sensitive to opioids. Instead of reporting these data as values in the text marked as “data not shown”, it would be useful to show them as an additional figure.

2- A conclusion sentence would be useful at the end of the last section of the results.

3- How was the stimulation intensity set for baseline responses? Were input/output curves quantified for both inputs? Was the stimulation intensity used for caudal and rostral inputs within a comparable range of the input/output curve? This is an important issue because in a variety of synapses the initial amplitude of a synaptic response influences the sign of plasticity that will be induced.

4- In Fig.3 A and B the time course of plasticity is quite different, with a sharp onset of potentiation in the absence of APV and a much slower onset in the presence of APV. How do the author interpret such effect?

5- The results in Fig 4 are puzzling. If stimulation of VGAT axons does not induce inhibitory plasticity, what would be the GABAergic afferents engaged by this plasticity?

Reviewer #2:

In this manuscript, the author(s) report that both high-frequency (HFS) and low-frequency (LFS) electrical stimulation of a region caudal to the VTA induced NMDA-independent LTP in putative VTA dopamine neurons. HFS-induced LTP was accompanied by decreases in the paired-pulse ratio (PPR) and 1/CV2 value, whereas the PPR and 1/CV2 value were not changed during LFS-LTP. These results appear to suggest that HFS-LTP is of presynaptic origin, and LFS-LTP is postsynaptic.

The authors hypothesized that “caudal” stimulation might bias the inputs to VTA differently than with “rostral” stimulation. However, the exact locations of “caudal” stimulation and “rostral” stimulation are not defined. Further, the authors speculate that structures located caudal to the VTA are affected by the caudal stimulation, but it is not clear whether these structures send projections through this region or through a different anatomical location. The authors should further discuss what specific axon tracts pass through or near the regions stimulated.

LFS-LTP is postsynaptic but it was not significantly altered by intracellular application of the calcium chelator EGTA or G-protein signaling blocker GDP-β-S and was not occluded by bath application of forskolin. The authors have not provided a mechanism for LFS-induced LTP and do not discuss potential mechanisms that might explain their results.

Other specific comments:

1. There is a large variability of evoked currents. This can be controlled by adjusting the stimulation intensity to evoke currents with similar magnitude. LTP can be differentially induced (or not induced) when current amplitudes (and neurotransmitter release probability) are of high vs. low magnitude. Did the authors note any differences in LTP induction with different baseline current amplitudes?

2. The high Cl- concentration in the pipet solution and absence of QX-314 may make it challenging to evoke currents without triggering action potentials that escape voltage clamping. Did the authors have any difficulty with this? Were action potential events observed, and if so, were they excluded from analysis?

3. The authors rarely provide mean values for different measurements in the figures or the text (e.g. for the results of Fig. 2B, 2D, 2F, and 2G). It is difficult to interpret the results when only p values and the 95% confidence interval are provided.

4. Related to this, it would be very useful if the authors showed the mean values in the Figures with before and after experiments (e.g. same figures as mentioned above).

5. Certain experiments have only very few cells. Specifically, Fig. 2D, 6C, and 6F. It is not possible to make convincing conclusions from n's of 5 or less.

6. The authors show different information in some figures than that described in the text. For example, Fig. 2F and 2G plot the PPR and I/CV2 values for cells that potentiated >10% of basal values, but this is not mentioned in the Results. The stats mentioned in the Results for Fig. 2G are not statistically significant, whereas they are in Fig. 2G. This is not appropriate and should be corrected.

7. The LFS-LTP does not follow the typical time course for LTP, in that it does not occur until ~15+ min after caudal LFS. The authors should discuss why this might occur and potential mechanisms for this.

## Author Response

Dear Dr. Castillo:

We would like to submit our revised manuscript (eN-NWR-0137-19) for consideration at eNeuro. We appreciate the extra time we were granted to get the new experiments completed, and have addressed the reviewers' comments point by point below in blue font. We hope that you will now find our paper acceptable for publication.

Response to the reviewers:

We are very grateful to both reviewers for their thoughtful and excellent suggestions for revision. We have taken these seriously and have added new data as well as text revision to the manuscript.

Reviewer #1:

The manuscript reports how different forms of inhibitory plasticity may be induced by different inputs to dopaminergic neurons in the VTA. While the manuscript does not identify specific mechanisms underlying such effects, it excludes a variety of signaling cascades that are often engaged in the induction of plasticity, pointing to a novel, yet to be discovered, set of mechanisms for inhibitory plasticity in this region. Overall, the experiments are well designed and the quality of the data is high. I have a few minor comments for the authors:

1- The effect of DAMGO on both rostral and caudal evoked responses is important to show that both inputs are equally sensitive to opioids. Instead of reporting these data as values in the text marked as “data not shown”, it would be useful to show them as an additional figure.

We have taken the reviewer's suggestion and added these data as panels E and F in Figure 1.

2- A conclusion sentence would be useful at the end of the last section of the results.

We have now added this sentence:

“Together our results suggest a mechanism that does not require postsynaptic GPCRs or Ca2+ influx. Future experiments will be needed to understand this novel form of LTP.”

3- How was the stimulation intensity set for baseline responses? Were input/output curves quantified for both inputs? Was the stimulation intensity used for caudal and rostral inputs within a comparable range of the input/output curve? This is an important issue because in a variety of synapses the initial amplitude of a synaptic response influences the sign of plasticity that will be induced.

We used input-output curves to identify the stimulation intensity used for plasticity experiments for both rostral and caudal afferents. The baseline amplitude was selected to be in the middle 50% of this generated curve. However, we did not find any correlation between stimulation intensity and LTP magnitude (see figures below). We have added this information to the methods.

4- In Fig.3 A and B the time course of plasticity is quite different, with a sharp onset of potentiation in the absence of APV and a much slower onset in the presence of APV. How do the author interpret such effect?

We observed both a slower onset of potentiation after LFS, although sometimes the potentiation occurred sooner. Shown below are two experiments, one without APV with a slower LTP onset, and an experiment with APV with an immediate onset. It is possible that in some experiments, or in some subset of dopamine neurons, a transient perhaps presynaptic depression follows the prolonged 1 Hz stimulation, producing the apparent slow onset LTP; in other cells or group of stimulated axons, this may be less pronounced. The depression could result from vesicle depletion, or from activation of mGluRs, or endocannabinoid or GABAB receptors by neurotransmitters released during LFS. Further work will be needed to tease this apart. For example when we know how to block the LTP, any residual depression may be more apparent.

5- The results in Fig 4 are puzzling. If stimulation of VGAT axons does not induce inhibitory plasticity, what would be the GABAergic afferents engaged by this plasticity?

We believe that inhibitory plasticity has indeed been engaged, but rather that two (or more) forms of plasticity were induced simultaneously at different GABAergic afferents on the same cell, leading to no net effect on IPSC amplitude. E.g., we know that LFS from a rostral stimulation site induces LTD that is independent of NMDARs (Dacher and Nugent, 2011; Neuropharmacol 61: 1166), and show here that caudal afferents undergo LTP with the same stimulus; the full-field light stimulation of all GABAergic inputs may simply sum these and yield no change from baseline values. Again, once we identify the underlying mechanisms, it should be possible to separate distinct mechanisms by blocking one or another. We have added this idea to introduce the experiment:

“There are many sources of GABAergic inhibition in the VTA and given that LFS can induce either LTD or LTP, depending on stimulation site, we wondered which form of plasticity was predominant when activating GABAergic synapses more globally. We hypothesized that just a subset of VTA synapses express LFS-LTPGABA, so that when using optical stimulation of VGAT+ inputs in a VGAT-ChR2 transgenic mouse line, both forms of plasticity might occur at different synapses on the same dopamine cell.”

Reviewer #2:

In this manuscript, the author(s) report that both high-frequency (HFS) and low-frequency (LFS) electrical stimulation of a region caudal to the VTA induced NMDA-independent LTP in putative VTA dopamine neurons. HFS-induced LTP was accompanied by decreases in the paired-pulse ratio (PPR) and 1/CV2 value, whereas the PPR and 1/CV2 value were not changed during LFS-LTP. These results appear to suggest that HFS-LTP is of presynaptic origin, and LFS-LTP is postsynaptic.

The authors hypothesized that “caudal” stimulation might bias the inputs to VTA differently than with “rostral” stimulation. However, the exact locations of “caudal” stimulation and “rostral” stimulation are not defined. Further, the authors speculate that structures located caudal to the VTA are affected by the caudal stimulation, but it is not clear whether these structures send projections through this region or through a different anatomical location. The authors should further discuss what specific axon tracts pass through or near the regions stimulated.

Very good point. We have improved our Figure 1A diagram, and included text referring to more specific locations of the recording electrodes in the methods and results. We now discuss our thoughts on what regions may be activated by a caudally-located electrode in the discussion section entitled, GABAergic afferents innervating the VTA:

“It is difficult to be certain precisely which afferents are sufficiently close to the caudal stimulation site to be activated by our stimulus protocol. Regions other than the RMTg that are located caudal to the VTA with reported GABAergic innervation include: the dorsal raphe, periaqueductal gray, pedunculopontine nucleus, and laterodorsal tegmentum (Beier et al., 2015, Faget et al., 2016, Omelchenko and Sesack, 2010, Ntamati et al., 2018). Given the placement of the caudal electrode in our experiments, it is possible that the presynaptic source of those inputs is from one of these caudal brain regions, although it is alternatively possible that regions located elsewhere in the brain send projections that pass through the caudal stimulation location.”

LFS-LTP is postsynaptic but it was not significantly altered by intracellular application of the calcium chelator EGTA or G-protein signaling blocker GDP-β-S and was not occluded by bath application of forskolin. The authors have not provided a mechanism for LFS-induced LTP and do not discuss potential mechanisms that might explain their results.

We are actively working to identify the mechanisms underlying LTP, and have expanded our discussion section to include more speculation as to the mechanism of LFS-LTP:

“We speculate that LFS with mild depolarization may lead to release of a signaling molecule from the post- or pre-synaptic cell. If different synapses express different receptor subtypes for that signaling molecule, then release via LFS could result in distinct synaptic strength changes. Future experiments will be needed to determine if HFS-LTP and LFS-LTP result e.g. from activation by metabotropic glutamate, endocannabinoid, or dopamine receptors.”

Other specific comments:

1. There is a large variability of evoked currents. This can be controlled by adjusting the stimulation intensity to evoke currents with similar magnitude. LTP can be differentially induced (or not induced) when current amplitudes (and neurotransmitter release probability) are of high vs. low magnitude. Did the authors note any differences in LTP induction with different baseline current amplitudes?

We did not note any differences based upon starting IPSC amplitude. See graphs below for correlation analysis.

Caudal or rostral HFS:

2. The high Cl- concentration in the pipet solution and absence of QX-314 may make it challenging to evoke currents without triggering action potentials that escape voltage clamping. Did the authors have any difficulty with this? Were action potential events observed, and if so, were they excluded from analysis?

We did not have difficulty with IPSCs evoking action potentials that escaped voltage clamping. On the rare occasion that a cell began spiking in voltage clamp, we excluded that cell from analysis entirely. We have added this information to the methods section.

3. The authors rarely provide mean values for different measurements in the figures or the text (e.g. for the results of Fig. 2B, 2D, 2F, and 2G). It is difficult to interpret the results when only p values and the 95% confidence interval are provided.

Thank you for this observation, we have now included mean values {plus minus} SEM where appropriate.

4. Related to this, it would be very useful if the authors showed the mean values in the Figures with before and after experiments (e.g. same figures as mentioned above).

We appreciate this suggestion, and have now added means to each of these figures.

5. Certain experiments have only very few cells. Specifically, Fig. 2D, 6C, and 6F. It is not possible to make convincing conclusions from n's of 5 or less.

As requested, we have now performed more experiments for the noted figures to increase n's; we have updated the figures and text accordingly.

6. The authors show different information in some figures than that described in the text. For example, Fig. 2F and 2G plot the PPR and I/CV2 values for cells that potentiated >10% of basal values, but this is not mentioned in the Results. The stats mentioned in the Results for Fig. 2G are not statistically significant, whereas they are in Fig. 2G. This is not appropriate and should be corrected.

Thank you for noticing this, we have corrected our errors.

7. The LFS-LTP does not follow the typical time course for LTP, in that it does not occur until ~15+ min after caudal LFS. The authors should discuss why this might occur and potential mechanisms for this.

See above regarding response to LFS-LTP mechanism and variability of LTP time course in reviewer #1, point 4. We favor the possibility that after the low-frequency stimulation at 1 Hz, there could be presynaptic depression of neurotransmitter release that masks the LTP, which could have a rapid onset (as occasionally seen in some experiments).
